# Petrogenesis and tectonic significance of the Baipu granite porphyry in the Huangtian uranium deposit, northeastern Guangdong, South China

**DOI:** 10.1371/journal.pone.0338050

**Published:** 2026-01-06

**Authors:** Kun Ruan, Jianyong Wu, Ziqiang Long, Zhuang Min

**Affiliations:** 1 Research Institute 290 CNNC, Shaoguan, China; 2 Jiangxi University of Water Resources and Electric Power, Nanchang, China; Universiti Teknologi Malaysia, MALAYSIA

## Abstract

The genetic link between Late Yanshanian granitic magmatism and uranium mineralization in South China remains a subject of active investigation, with the petrogenesis and tectonic drivers of many uranium-hosting plutons being poorly constrained. To address this knowledge gap, we present an integrated study of the Baipu granitic porphyry in the Huangtian deposit, Northeast Guangdong, incorporating petrographic observations, zircon U–Pb geochronology, and whole-rock geochemistry. Our results show that the pluton was emplaced at 159.4 ± 1.4 Ma and is classified as a high-silica, potassic, strongly peraluminous S-type granite. It exhibits significant enrichment in LREEs and incompatible elements (e.g., Rb, Th, U), coupled with pronounced negative Eu and Sr anomalies. These geochemical signatures indicate derivation from the partial melting of psammitic crustal sources, with limited fractional crystallization, in a post-collisional setting triggered by Late Jurassic lithospheric delamination. We conclude that the Baipu porphyry is not merely spatially associated but is genetically linked to uranium mineralization, serving as both a metal source and a heat engine for ore-forming hydrothermal systems. This model underscores the high exploration potential for uranium deposits associated with S-type granites in similar extensional tectonic settings across South China.

## 1. Introduction

Northeastern Guangdong is a crucial uranium and polymetallic metallogenic belt in South China and has long been a focus of geological research [1–4]. Since the discovery of the Huangtian uranium deposit in the 1950s, studies have primarily focused on its metallogenic conditions, ore–controlling factors, and exploration potential [5]. Early work emphasized mineralization characteristics, wall–rock alteration, and ore–controlling structures [6], establishing a broad genetic link between uranium mineralization and Yanshanian granites [7], though with limited attention to the granite porphyry within the ore–hosting intrusion.

Since the 1990s, the application of high–precision dating techniques (e.g., SHRIMP and LA–ICP–MS zircon U–Pb dating; [8–10]) has enabled the construction of a refined chronological framework forgranites in South China’s granites. These advances confirmed that uranium mineralization mainly occurred from the Late Jurassic onward (140 ~ 60 Ma; [11]), associated with magmatic–hydrothermal activities under the subduction of the Paleo–Pacific Plate [12].

Recent metallogenic models further suggest that uranium enrichment is influenced not only by magma source characteristics [13,14] but also by post–magmatic hydrothermal alteration and regional tectonic settings [15,16]. The Baipu granite porphyry, which hosts part of the Huangtian deposit, shows elevated uranium background values (>10 ppm) and intense alteration, indicating multi–stage magma-fluid interactions [6]. Nevertheless, systematic studies on its petrogenesis and tectonic setting remain limited, and comparative analyses with contemporaneous regional granites is sparse, constraining a deeper understanding of its geodynamic context and metallogenic significance.

While the uranium potential of this region is well recognized, a detailed petrogenetic and geochronological investigation of the host Baipu granite porphyry has been lacking, leaving its precise tectonic setting and role in mineralization poorly constrained. This study focuses on the granite porphyry from the Huangtian uranium deposit. By integrating LA–ICP–MS zircon U–Pb dating for high–precision geochronology with whole–rock major and trace element analyses, we systematically determine its formation age, material sources, and magmatic evolution. The results not only offer a theoretical foundation for regional uranium exploration but also provide new insights into the mechanisms of large–scale uranium enrichment during the Late Mesozoic tectonic transition in South China.

## 2. Regional geological background

The study area is tectonically located within the Yuedong Indosinian Fold Belt of the Cathaysia Block, South China, situated at the intersection of the southwestern segment of the NE–trending Wuyi–Heyuan metallogenic belt and the eastern segment of the EW trending Nanling metallogenic belt, representing a long–term active zone of tectonic, magmatic, and polymetallic mineralization [17]. The region features well–developed structures, with regional faults converging in the NE trending Heyuan–Jianyang deep fault zone, Dengta–Xunwu deep major fault, NW trending Lufeng–Chenzhou major fault zone, and EW trending Fogang–Fengliang major fault zone. The stratigraphic sequence is relatively complete, primarily consisting of Cambrian Bacun Group low–grade metamorphic rocks, Jurassic–Triassic fine–grained sandy conglomerates, and Upper Cretaceous Nanxiong Group purplish–red sandy conglomerates interbedded with calcareous sandstones [6].

**Fig 1:**
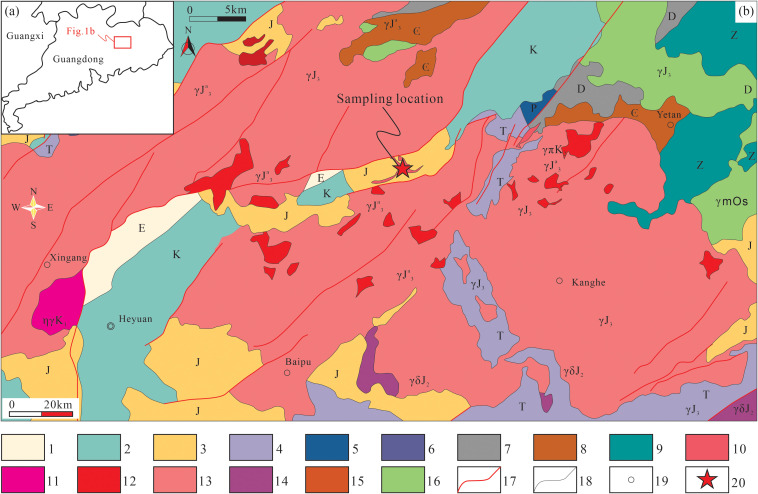
Geological Sketch of uranium deposit in Huangtian area, Heyuan City, Guangdong Province (adapted from [7]). **(a)** Tectonic location of the study area within the South China; **(b)** Simplified geological map of the Huangtian area showing the distribution of the Baipu granite porphyry and sample locations. 1. Paleogene; 2. Cretaceous; 3. Jurassic; 4. Triassic; 5. Permian; 6. Carboniferous; 7. Devonian; 8. Cambrian; 9. Aurora; 10. Late Cretaceous granitic porphyry; 11. Early Cretaceous biotite monzogranite; 12. Late Jurassic meso–grained biotite monzogranite; 13. Late Jurassic medium–grained biotite granite; 14. Middle Jurassic granodiorite; 15. Ordovician Silurian mixed rocks; 16. diabase gabbro; 17. Fault; 18. Geological boundaries; 19. Town/City; 20. Sampling location.

The Baipu granite pluton, with an outcrop area of approximately 888 km^2^, forms the eastern extension of the southern Nanling granitic belt (Fogang pluton). It is primarily composed of biotite granite as the dominant lithology and later–stage granite porphyry intrusions (Fig 1). The pluton contains NE, NW, and SN–trending fault systems. The Huangtian uranium deposit is located in the inner contact zone and western tongue–like body at the northern margin of the Baipu pluton, hosted mainly in medium to coarse grained porphyritic biotite granite across an area of about 2 km^2^, controlled by the structural junction of the EW trending Yihuang and NE trending Liushe tectonic zones. Uranium orebodies predominantly occur as concealed veins, lenses, and lenticles within the transitional facies of the porphyritic biotite granite, surrounded by extensively altered fine grained two–mica granite, albitized medium–fine grained granite apophyses, diorite, and granite porphyry dikes. The mineralized rocks include altered cataclastic granite, lamprophyre, and granite porphyry. The ore types are dominated by altered cataclastic granite–type, followed by altered cataclastic lamprophyre–type and altered cataclastic granite porphyry–type. The primary uranium minerals are pitchblende, with subordinate uraninite and autunite, while the gangue minerals consist of quartz, calcite, fluorite, sericite, and chlorite.

## 3. Petrographic characteristics

**Fig 2:**
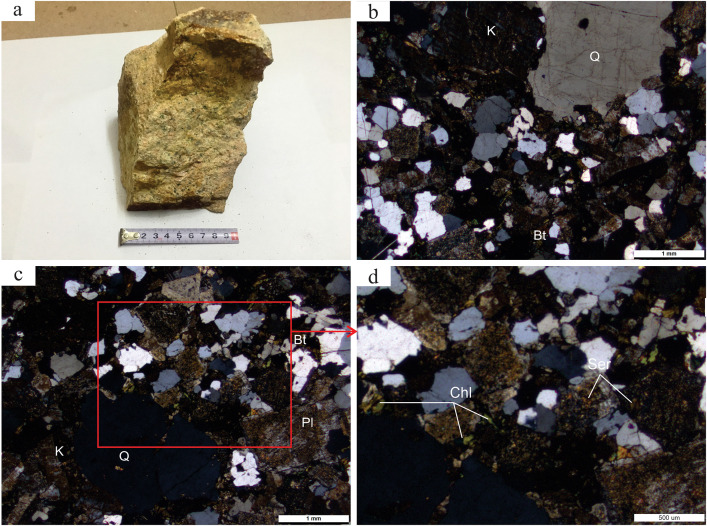
Hand specimens (a) and microscopic photographs (b, c, d) of Baipu granite porphyry. **(a)** Hand specimens of the Baipu granite porphyry. **(b)** Feldspar and quartz phenocrysts in microscopic images. **(c)** granitic texture in microscopic images. **(d)** The sericite and chlorite metamorphoses of feldspar in microscopic images. Q–Quartz; Bt–biotite; K–potassium feldspar; Pl–plagioclase; Ser–sericite; Chl–chlorite.

The five granite porphyry samples collected from the Huangtian uranium deposit exhibit a pale yellow color (Fig 2a) with porphyritic texture and alteration mineralogy (e.g., hematitization, clay alteration). Phenocrysts (20 ~ 25% in volume) consist of quartz, K–feldspar, plagioclase and minor biotite (Fig 2b). Quartz (2 ~ 5%) occurs as anhedral grains (2 ~ 3 mm) with weak resorption textures. K–feldspar (12 ~ 17%) displays euhedral to subhedral tabular forms with grid twinning and perthitic texture, containing fractures filled by calcite and quartz (Fig 2c). Plagioclase (3 ~ 7%) shows tabular morphology with polysynthetic twinning. Scattered biotite flakes exhibit pale yellow to yellowish–green pleochroism and weak chloritization (Fig 2d). The matrix (75 ~ 80%) displays microgranitic texture composed mainly of quartz (30%), feldspar (40%) and biotite (5%).

## 4. Zircon U–Pb age dating

The granite porphyry samples were crushed and processed through mineral separation, hand–picking, and mounting procedures. Cathodoluminescence (CL) imaging was performed using scanning electron microscopy to select smooth, inclusion–free, and homogeneous zircon domains for analysis. LA–ICP–MS zircon U–Pb dating and whole–rock major and trace element analyses were conducted at Wuhan SampleSolution Analytical Technology Co., Ltd. The analytical system consisted of an Agilent 7700e ICP–MS coupled with a COMPexPro 102 ArF 193 nm excimer laser and MicroLas optical system, using a 30 μm beam diameter. Zircon standard 91500 and glass reference material NIST610 were employed as external standards for U–Pb isotopic fractionation correction. Two standard measurements were conducted per 12 analytical spots. Signal processing and isotopic ratio calculations were performed using GLITTER 4.0, with common Pb correction via Andersen’s method. Age calculations and concordia diagrams were generated using Isoplot v.3.6 [18]. The U–Pb isotopic data and results are presented in Table 1.

**Table 1:** LA-ICP-MS zircon U-Pb age of granite porphyry.

Point ID	Th	U	Th/U	^207^Pb/^206^Pb	1σ	^207^Pb/^235^U	1σ	^206^Pb/^238^U	1σ	^206^Pb/^238^U	1σ
1	1914	4437	0.43	0.05948	0.00127	0.21425	0.00411	0.02612	0.00025	166	2
2	795	1659	0.48	0.06098	0.00307	0.21628	0.01066	0.02572	0.00026	164	2
3	695	2161	0.32	0.05004	0.00170	0.17562	0.00582	0.02546	0.00019	162	1
4	1126	6463	0.17	0.04935	0.00020	0.17675	0.00119	0.02602	0.00023	166	1
5	276	663	0.42	0.04884	0.00017	0.16960	0.00122	0.02519	0.00018	160	1
6	818	2765	0.3	0.04808	0.00009	0.16659	0.00132	0.02512	0.00019	160	1
7	575	1596	0.36	0.04882	0.00018	0.16787	0.00128	0.02492	0.00015	159	1
8	1924	5872	0.33	0.05693	0.00225	0.22155	0.00825	0.02822	0.00038	179	2
9	591	1207	0.49	0.05688	0.00109	0.19531	0.00339	0.02490	0.00020	159	1
10	696	1710	0.41	0.05124	0.00094	0.16265	0.00269	0.02302	0.00018	147	1
11	370	947	0.39	0.05067	0.00019	0.17678	0.00155	0.02531	0.00023	161	1
12	706	2408	0.29	0.04904	0.00011	0.16861	0.00122	0.02493	0.00018	159	1
13	1128	6080	0.19	0.05225	0.00032	0.18293	0.00250	0.02545	0.00036	162	2
14	435	1082	0.4	0.04828	0.00014	0.16775	0.00140	0.02519	0.00019	160	1
15	586	1988	0.29	0.05049	0.00015	0.17647	0.00157	0.02533	0.00019	161	1
16	1278	5003	0.26	0.04831	0.00014	0.16881	0.00153	0.02532	0.00020	161	1
17	1456	4701	0.31	0.05450	0.01135	0.17224	0.03568	0.02292	0.00046	146	3
18	691	1817	0.38	0.05046	0.00035	0.17334	0.00211	0.02487	0.00019	158	1
19	1210	5928	0.2	0.04906	0.00086	0.17219	0.00262	0.02546	0.00022	162	1
20	957	2343	0.41	0.04960	0.00110	0.16700	0.00353	0.02442	0.00016	156	1
21	660	1122	0.59	0.04960	0.00069	0.16961	0.00163	0.02452	0.00015	156	1
22	689	3810	0.18	0.05016	0.00011	0.17254	0.00105	0.02495	0.00016	159	1
24	1022	8764	0.12	0.04938	0.00080	0.16595	0.00256	0.02438	0.00011	155	1

**Fig 3:**
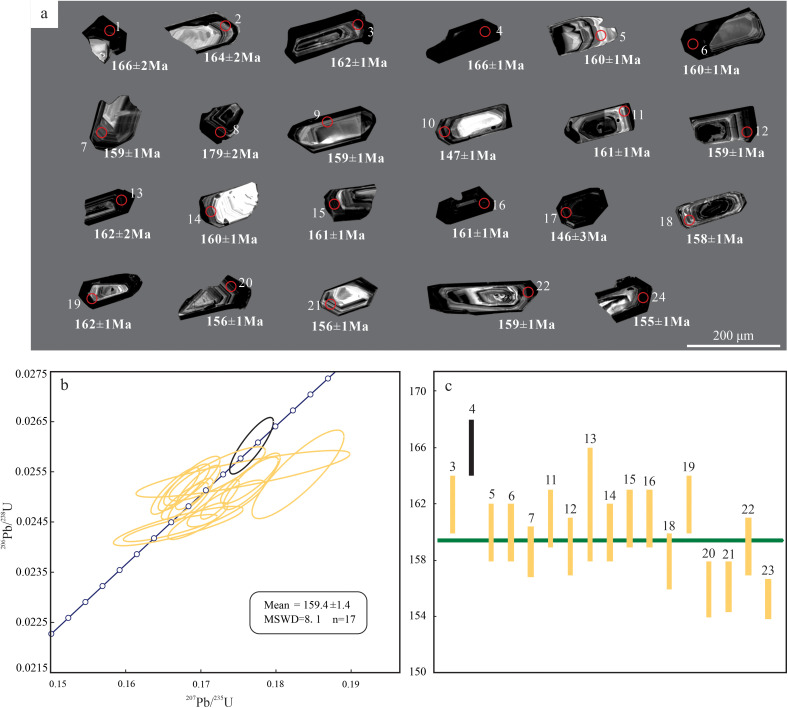
LA–ICP–MS zircon U–Pb dating for Baipu granite porphyry. **(a)** Cathodoluminescence images. **(b)** U–Pb concordia diagram. **(c)** ^206^Pb/^238^U weighted average diagram.

**Fig 4:**
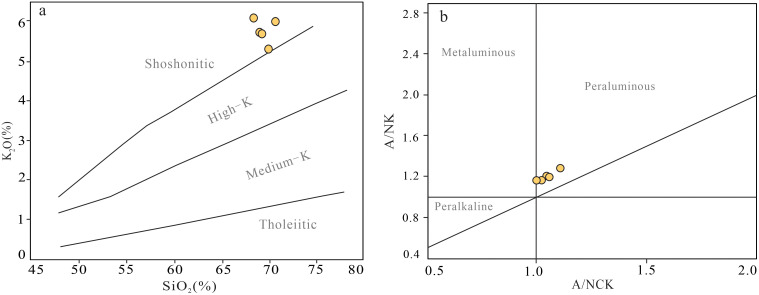
Diagram of major elements for granite porphyry of Baipu. **(a)** SiO_2_–K_2_O diagram (adapted from [20]); **(b)** A/NCK–A/NK diagram. (adapted from [21]).

Zircons from the granite porphyry are predominantly prismatic to stubby (Fig 3), exhibiting euhedral to subhedral morphologies with grain sizes of 80 ~ 200 μm and aspect ratios of 1:1 ~ 4:1. Th/U ratios range from 0.12 to 0.59 (average 0.34, > 0.1), and CL images display typical magmatic oscillatory zoning, confirming a magmatic origin [19]. The zircons contain high U (663 ~ 8764 ppm, average 3240 ppm) and Th (276 ~ 1924 ppm, average 896 ppm) concentrations, with darker rims likely reflecting U enrichment. Of 23 analytical spots, five discordant points (possibly due to U–Pb system disturbance) were excluded. The remaining 18 concordant spots cluster on/near the concordia curve (Fig 3), yielding ^206^Pb/^238^U ages of 147 ~ 166 Ma. The weighted mean age of 159.4 ± 1.4 Ma (MSWD = 8.1, n = 18) represents the emplacement age of the granite porphyry, constraining its formation to the Late Jurassic (Fig 4).

## 5. Geochemical characteristics

Petrographic observations under microscopy guided the selection of five granite porphyry samples from the study area for major and trace element analyses. Major elements were determined using a Rigaku Primus II X–ray fluorescence spectrometer (XRF) after sample fusion with NH_4_NO_3_ oxidizer at 1150°C. Trace and rare earth elements were analyzed by acid digestion followed by measurement with an Agilent 7700e ICP–MS, achieving analytical precisions of 1% and 5% respectively [22]. Complete analytical results and calculated parameters are presented in Table 2.

**Table 2:** Major (wt%) and trace elements (10^−6^) compositions of the granite.

Number	4018005	4018006	4018007	4018008	4018009
SiO_2_	68.51	69.16	70.07	69.77	69.10
FeO	0.15	0.21	0.26	0.13	0.18
Fe_2_O_3_	2.92	2.95	1.94	2.13	2.81
Al_2_O_3_	14.93	14.56	14.48	14.69	14.31
CaO	1.68	1.24	1.360	1.350	1.58
MgO	0.726	0.773	0.919	0.981	0.756
MnO	0.064	0.065	0.060	0.056	0.064
TiO_2_	0.347	0.360	0.386	0.397	0.353
P_2_O_5_	0.095	0.099	0.105	0.100	0.097
K_2_O	6.04	5.68	5.27	5.97	5.71
Na_2_O	2.12	2.36	2.19	2.36	2.40
LOI	1.97	2.42	1.36	1.46	2.50
Total	99.57	99.90	98.43	99.41	99.88
A/CNK	1.02	1.05	1.10	1.01	0.99
A/NK	1.20	1.19	1.28	1.16	1.16
CaO/Na_2_O	0.79	0.52	0.62	0.57	0.66
K_2_O/Na_2_O	2.85	2.400	2.410	2.53	2.380
Na_2_O+K_2_O	8.16	8.04	7.46	8.33	8.11
Rb	264.00	233.00	159.70	173.80	275.00
Sr	109.40	116.40	94.20	128.60	145.10
Y	18.0	19.7	23.8	21.6	19.6
Zr	76.10	74.00	77.80	75.50	84.60
Hf	2.69	2.52	2.85	2.81	2.99
Nb	13.80	12.20	10.90	12.50	14.40
Ta	1.29	1.09	0.87	1.10	1.40
Ba	513.00	503.00	494.10	548.90	547.00
Th	41.20	40.60	40.90	39.00	45.00
U	2.37	2.55	2.24	2.14	2.75
Th/U	17.38	15.92	18.26	18.22	16.36
Rb/Sr	2.41	2	1.7	1.35	1.9
Rb/Ba	0.51	0.46	0.32	0.32	0.5
Rb/Nb	19.13	19.1	14.65	13.9	19.1
Nb/Ta	10.7	11.19	12.53	11.36	10.29
Zr/Hf	28.29	29.37	27.3	26.87	28.29
La	67.8	58.3	79.3	57.5	81.9
Ce	120	110	239	162	146
Pr	13.7	12.1	15.8	11.6	16.3
Nd	48.6	43.3	52.9	39.2	57.4
Sm	7.93	7.43	8.52	6.58	9.45
Eu	0.96	0.82	0.61	0.49	1.01
Gd	6.74	6.29	7.24	5.87	7.72
Tb	0.86	0.79	0.87	0.72	1
Dy	4.6	4.08	4.43	3.98	5.06
Ho	0.79	0.77	0.79	0.74	0.97
Er	2.22	2.13	2.33	2.11	2.54
Tm	0.32	0.29	0.32	0.3	0.37
Yb	1.96	1.84	2.04	1.84	2.17
Lu	0.27	0.23	0.31	0.29	0.31
ΣREE	276.75	248.37	414.46	293.22	332.2
LREE	258.99	231.95	396.13	277.37	312.06
HREE	17.76	16.42	18.33	15.85	20.14
LREE/HREE	14.58	14.13	21.61	17.5	15.49
δEu	0.4	0.37	0.24	0.24	0.36
(La/Yb)_N_	21.12	21.41	26.27	21.12	25.5
(La/Sm)_N_	5.38	4.94	5.86	5.5	5.46
(Gd/Yb)_N_	2.79	2.77	2.88	2.59	2.88
δCe	0.92	0.97	1.58	1.47	0.94

### 5.1. Major elements

**Fig 5:**
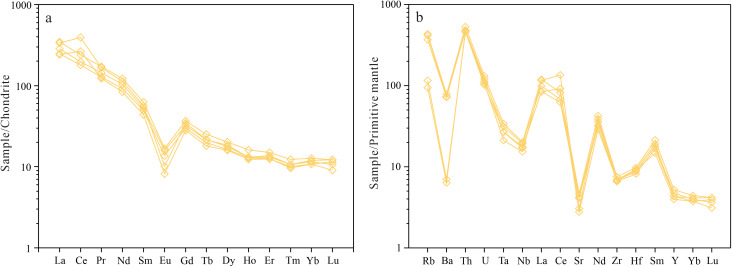
The REE and trace element of granite porphyry. **(a)** The REE distribution patterns (normalized values from [23]). **(b)** The trace element spider diagram (normalized values from [24]).

The granite porphyry exhibits SiO_2_ contents of 68.51 ~ 70.07 wt.% and Al_2_O_3_ contents of 14.31 ~ 14.93 wt.%. Total alkalis (Na_2_O+K_2_O = 7.46 ~ 8.33 wt.%) show potassium enrichment (K_2_O/Na_2_O=2.38 ~ 2.85), classifying the rocks as subalkaline granites. In the SiO_2_–K_2_O diagram (Fig 5a), samples plot within the shoshonitic series field, displaying strongly peraluminous characteristics (A/CNK = 0.99 ~ 1.10, average 1.03; Fig 5b). The rocks contain low CaO (1.24 ~ 1.68 wt.%), TFe_2_O_3_ (2.23 ~ 3.19 wt.%), MgO (0.73 ~ 0.98 wt.%) and P_2_O_5_ (0.09 ~ 0.11 wt.%) concentrations.

### 5.2. Trace elements

Total REE contents (∑REE = 248.37 ~ 414.46 ppm, average 313.00 ppm) exceed upper continental crust values (210.3 ppm; [25]). The LREE/HREE ratios (14.13 ~ 21.16, average 16.66) and (La/Yb)_N_ values (21.12 ~ 26.27, average 23.08) indicate significant LREE enrichment and fractionation. The chondrite–normalized REE pattern shows a right–inclined trend (Fig 5a) with pronounced negative Eu anomalies (δ_Eu_ = 0.24 ~ 0.40, average 0.32). The coupled decrease in LREE/HREE ratios and δEu is attributed to the fractional crystallization of plagioclase and LREE-rich accessory phases (e.g., apatite, allanite), complemented by the residual stability of HREE-retaining garnet in the source during partial melting.

The rocks are enriched in large–ion lithophile elements (Rb = 159.70 ~ 275.00 ppm; Th = 39.00 ~ 45.00 ppm; U = 2.14 ~ 2.75 ppm) but depleted in Ba (494.10 ~ 548.90 ppm), Sr (94.20 ~ 145.10 ppm) and Nb (10.90 ~ 14.40 ppm), forming characteristic “V”–shaped troughs in spider diagrams (Fig 5b). Low Nb/Ta ratios (10.29 ~ 12.53, average 11.21) relative to mantle–derived magmas (Nb/Ta = 17) indicate negligible mantle contribution. Depletions in Ba, Sr and Nb likely reflect fractional crystallization of Ti–bearing minerals (e.g., ilmenite, sphene) and apatite. High Rb/Sr ratios (1.35 ~ 2.41, average 1.87) confirm extensive magmatic differentiation [26].

## 6. Discussions

### 6.1. Geochemical evidence for a peraluminous s-type granite

**Fig 6:**
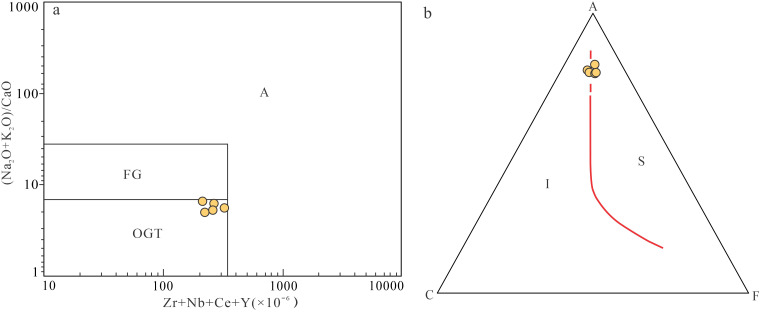
Discriminant diagram of rock genetic type. **(a)** The (Zr + Nb + Ce + Y)×10^−6^-(Na_2_O+K_2_O)/CaO diagram (adapted from [27]). **(b)** The ACF diagram (adapted from [28]). OGT–undifferentiated I, S and M–type granites; FG–differentiated granite; a–A type granites.

The granite porphyry exhibits high silica and alkali contents with K_2_O > Na_2_O, along with elevated Rb/Sr ratios and pronounced negative Eu anomalies, indicating significant fractional crystallization. In the (Zr + Nb + Ce + Y)–(Na_2_O+K_2_O)/CaO diagram (Fig 6a), samples plot within the unfractionated granite field, while the absence of characteristic alkaline mafic minerals precludes classification as A–type granite. Furthermore, typical M–type granites have low K_2_O contents (<1 wt.%), whereas the studied samples show much higher average K_2_O values (5.73 wt.%) and lack amphibole, eliminating an M–type affinity. Strongly peraluminous I-type granites often show a decrease in P_2_O_5_ with increasing SiO_2_ due to apatite fractionation, whereas S-type granites typically have high P_2_O_5_. The P_2_O_5_ content in our samples is consistent with an S-type origin (Zhu et al., 2009, [29,30]). In the ACF diagram (Fig 6b), all samples fall within the S–type granite field, exhibiting geochemical signatures (Rb, Th, U, Sm enrichment; Ba, Sr, Ti depletion) consistent with typical S–type granites in South China [31]. These features collectively identify the granite porphyry as a moderately fractionated S–type granite.

The granite porphyry shows Rb/Sr ratios (1.35 ~ 2.41) significantly higher than eastern China’s upper crust average (0.32; [32]), Zr/Hf ratios (26.87 ~ 29.37) below the upper crustal average (37), and Rb/Nb ratios (5.48 ~ 19.13) exceeding global upper crust values (4.5; [23]). These characteristics confirm a crustal origin through partial melting of sedimentary sources [31].

### 6.2. Source constraints: Crustal anatexis of Paleoproterozoic rocks

**Fig 7:**
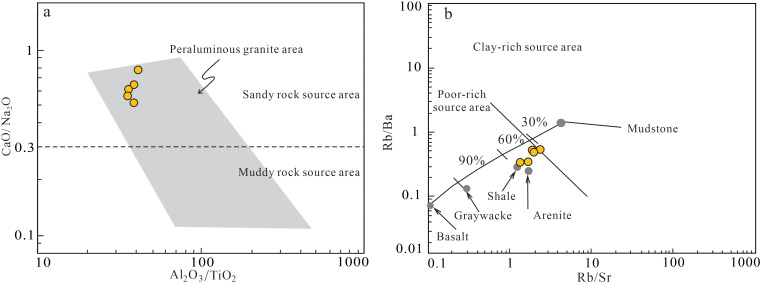
Discrimination map of magma source area (adapted from [33]). **(a)** The (Al_2_O_3_/TiO_2_)-(CaO/Na_2_O) diagram. **(b)** The (Rb/Sr)/(Rb/Ba) diagram.

Following Sylvester's [33] methodology using CaO/Na_2_O ratios and TFeO + MgO + TiO_2_ contents, the granite porphyry shows CaO/Na_2_O=0.52 ~ 0.79 (average 0.63, > 0.3) and TFeO + MgO + TiO_2_ = 3.54 ~ 4.32 wt.% (average 4.01 wt.%, > 4 wt.%). The relatively high CaO/Na_2_O and low Rb/Sr ratios, combined with Al_2_O_3_/TiO_2_–CaO/Na_2_O and Rb/Sr–Rb/Ba systematics (Fig 7b), suggest derivation from clay–poor psammitic sources, likely Paleoproterozoic metasedimentary rocks [34]. Source heterogeneity may reflect varying melting depths [26,35].

### 6.3. Tectonic setting: Asthenospheric upwelling and lithospheric thinning

Mesozoic Nanling granites exhibit predominant E–W trends, divisible into three magmatic belts from north to south: Zhuguang–Qingzhang, Dadong–Guidong, and Fogang–Xinfengjiang. This orientation contradicts expected NW–NNW trends from Paleo–Pacific subduction, indicating additional tectonic controls. Recent identification of Indosinian granites reveals Tethyan tectonic influences, evidenced by E–W mantle structures (Yuebei depression–Fogang slope–Guangzhou high) and gravity gradients.

Precise dating of the Baipu pluton’s granite porphyry (159.4 ± 1.4 Ma) reveals Early Yanshanian emplacement. Its high–K calc–alkaline composition suggests magma generation related to prior subduction, forming during post-collisional lithospheric delamination [36]. Regional studies document Indosinian (258 ~ 192 Ma) crustal thickening followed by Early Yanshanian (176 ~ 179 Ma) A–type granites, bimodal volcanism (158 ~ 179 Ma), and OIB–like basalts–clear evidence of asthenospheric upwelling and lithospheric thinning. These features collectively indicate an intraplate setting controlled by post–orogenic collapse.

**Fig 8:**
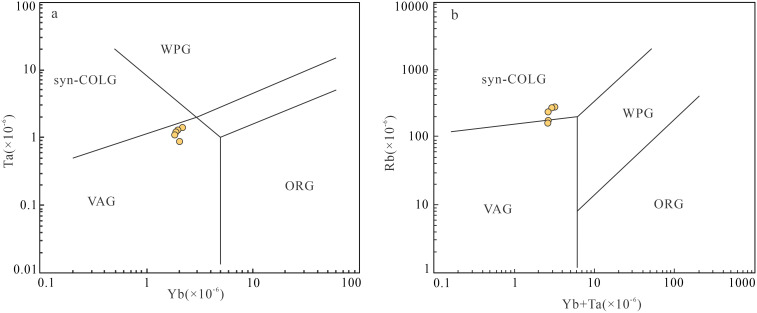
Discrimination diagram of Petrogenesis tectonic environment. **(a)** The Yb(×10^−6^)-Ta(×10^−6^) diagram. **(b)** The (Yb + Ta)(×10^−6^)-Rb(×10^−6^) diagram. Syn–COLG. syn–collision granite; WPG. in–plate granite; VAG. volcanic arc granite; ORG. Mid–ocean ridge granite.

In Pearce’s [37] tectonic discrimination diagrams (Fig 8), samples mainly plot in syn–collisional granite fields, overlapping with post–collisional/intraplate boundaries. Syn–collisional granites share characteristics with post–collisional types [38], consistent with Late Jurassic northern Guangdong’s extensional regime. Although strong HFSE (Nb, Ta, Zr, Hf) depletion typifies subduction–related magmas, the Baipu pluton’s intraplate setting suggests inherited source signatures rather than active subduction, as crustal melts typically show low HFSE contents.

**Fig 9:**
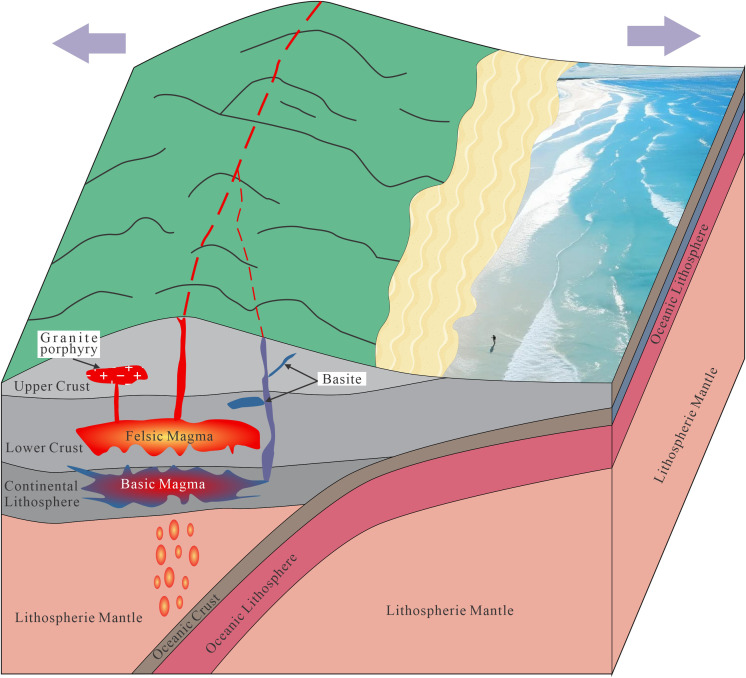
The genetic model diagram of Baipu granite porphyry.

Early Yanshanian post–orogenic extension may have triggered asthenospheric upwelling and lithospheric thinning, which in turn facilitated mafic magma underplating. Crustal melting–facilitated by fault–induced decompression and heat from mafic magmas generated felsic melts [39]. Hybridization of mafic and felsic magmas in deep chambers, followed by fractional crystallization, produced the granite porphyry (Fig 9).

## 7. Conclusion

The granite porphyry in the Huangtian uranium deposit was emplaced during the Late Jurassic, as constrained by a zircon U–Pb age of 159.4 ± 1.4 Ma. Geochemically, it is characterized by high silica content, alkali enrichment, and elevated K₂O over Na₂O, classifying it as a shoshonitic, peraluminous S-type granite. The rock exhibits significant enrichment in Rb, Th, U, and Sm, coupled with depletions in Ba, Sr, and Nb. It also shows light rare earth element (LREE) enrichment and a pronounced negative Eu anomaly. These features collectively indicate that the granite porphyry formed in a post-collisional intraplate extensional setting linked to the subduction of the Paleo-Pacific Plate, originating from partial melting of clay-poor sandy sedimentary rocks within a Late Jurassic crustal anatexis background.
